# HPV Infection: Immunological Aspects and Their Utility in Future Therapy

**DOI:** 10.1155/2013/540850

**Published:** 2013-08-20

**Authors:** Efthimios Deligeoroglou, Aikaterini Giannouli, Nikolaos Athanasopoulos, Vasileios Karountzos, Anastasia Vatopoulou, Konstantinos Dimopoulos, George Creatsas

**Affiliations:** ^1^Division of Pediatric-Adolescent Gynecology and Reconstructive Surgery, 2nd Department of Obstetrics and Gynecology, Athens University, Medical School, Aretaieion Hospital, Vassilisis, Sofias Avenue 76, 11528 Athens, Greece; ^2^Division of Pediatric and Adolescent Gynecology, 1st Department of Ob/Gyn Papageorgiou Hospital, University of Thessaloniki, Medical School, Perifereiaki Odos Thessalonikis-N, Efkarpias, 564 29 Thessaloniki, Greece

## Abstract

High prevalence and mortality rates of cervical cancer create an imperative need to clarify the uniqueness of HPV (Human Papillomavirus) infection, which serves as the key causative factor in cervical malignancies. Understanding the immunological details and the microenvironment of the infection can be a useful tool for the development of novel therapeutic interventions. Chronic infection and progression to carcinogenesis are sustained by immortalization potential of HPV, evasion techniques, and alterations in the microenvironment of the lesion. Inside the lesion, Toll-like receptors expression becomes irregular; Langerhans cells fail to present the antigens efficiently, tumor-associated macrophages aggregate resulting in an unsuccessful immune response by the host. HPV products also downregulate the expression of microenvironment components which are necessary for natural-killer cells response and antigen presentation to cytotoxic cells. Additionally HPV promotes T-helper cell 2 (Th2) and T-regulatory cell phenotypes and reduces Th1 phenotype, leading to suppression of cellular immunity and lesion progression to cancer. Humoral response after natural infection is inefficient, and neutralizing antibodies are not adequate in many women. Utilizing this knowledge, new endeavors, such as therapeutic vaccination, aim to stimulate cellular immune response against the virus and alter the milieu of the lesion.

## 1. Introduction

All sexually active individuals are liable to HPV infection during sexual intercourse. It is assessed that the risk of sexually active women to be infected sometime in their life is nearly 80% [1]. HPV infection alone is not adequate for the advancement to cervical cancer and other risk conditions such as smoking, prolonged oral contraception consumption, coinfections, and multiparity, immune-related diseases appear to lead the infection on the route of carcinogenesis [2–5]. The vast majority (90%) of HPV infections are cleared by the patients' immune system in three-year followup, whereas from the 10% that become chronic only 1% result in cervical cancer. The infection is usually clinically silent with absence of common genital symptoms, but it can be manifested with a spectrum of lesions from genital warts to invasive cancer [6]. Suppression of host immunity, persistence of the infection, and integration of the virus into the host DNA help a low grade squamous intraepithelial lesion (LSIL) to step up to high grade squamous intraepithelial lesion (HSIL) and even to invasive carcinoma of the cervix [7].

## 2. Materials and Methods

We scrutinized the current literature, using PubMed as our primary search database in order to explore the newest findings regarding specific aspects of HPV infection, including human immune response or immune tolerance and the route to carcinogenesis. Additionally, during our search, special consideration has been given to the established results of preventive vaccination and the cutting edge field of therapeutic vaccination. 

## 3. Results and Discussion

### 3.1. The Virus, the Genes, and the Proteins

More than 180 types of human papillomaviruses are known, and more are presumed to exist [8]. About 40 types of HPV belong to the alpha genus and affect squamous epithelium of skin and mucosal epithelium of anogenital region, and 15 of them can lead to cervical cancer [9]. Among HPV types, HPV16 and HPV18 are accountable for approximately 70% of cervical cancers around the world. The virus is 52–55 nm in diameter, surrounded by a proteinaceous coat, which forms an icosahedral capsid. HPV DNA is double-stranded, with a molecular weight of 5 × 10^6^ Da and length of 7900 base pairs, arranged in a circle [10]. HPV requires basal cells of the squamous epithelium, metaplastic cells of the squamocolumnar junction of the cervix, or rarely glandular cells of the endocervix in order to complete its life cycle [11]. Only basal cells are appropriate because coordination with the differentiation of keratinocytes is needed for successful virus multiplication. Initially viral DNA appears as an episome, not integrated in the host genetic material.

HPV genome consists of 8 open reading frames, 6 early genes (E1, E2, E4, E5, E6, and E7), and 2 late genes (L1, and L2), whose products vary from plain capsid proteins to immortalization tools, and a long control region (LCR). Early genes are expressed in the basal, suprabasal, and intermediate cells of the cervix, whereas the late genes, responsible for the capsid proteins, are activated in the apical strata. E1 prepares the viral genome to be replicated by the host replication machinery. E2 maintains the episomal form of the viral genome and organizes its transcription. E4 full potential is yet to be clarified. So far its expression is apparent throughout the epithelium. E4 facilitates viral replication and disrupts the cytoskeleton in order to facilitate the escape of the virions out of the differentiated cells. E5 protein, when present, modifies the function of growth factor receptors [12]. E6 and E7 are the major oncogenic tools in the viral genome. In low risk HPV types E6 and E7 appear inactive or weakly active. On the contrary the oncogenes in high risk type are transcribed as long as high risk HPV viruses integrate their DNA into the cellular DNA [13, 14]. E6 product binds mainly to tumor suppressor protein p53 and arrests the cell in the S phase of the cell cycle, hindering apoptosis and enhancing the transformation capability. E7 binds and inactivates tumor suppressor pRB resulting in tumorigenesis [14, 15].

### 3.2. Mechanism of HPV Infection

**Figure 1:**
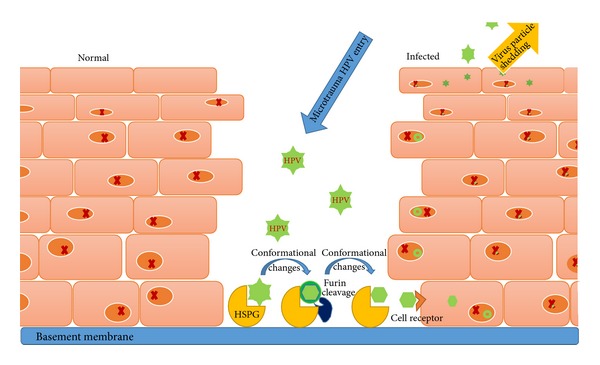
HPV reaches the basement membrane of the cervix through microtrauma. Interaction with HSPG via L1 protein induces conformational changes on the virus capsid, followed by furin cleavage and revealing of the binding site for the cell receptor.

In vivo, the virus does not bind directly to the cells, but it requires contact with the basement membrane. This contact can be accomplished by microabrasions in the cervical surface which reveal the basement membrane. The most plausible receptor of the major capsid protein (L1) seems to be the tissue-specific heparin sulfate proteoglycan (HSPG) which belongs to the glycosaminoglycan family [16–18]. HSPG can be found on both basement membrane and basal cells. The transfer between basement membrane and basal cells of the stratified epithelium is yet to be clarified. In vitro studies suggest another candidate receptor, laminin-5, which resides on the extracellular matrix [19]. On the other hand, other studies suggest that HPV binds directly to the basal cells [20]. Additionally to the aforementioned L1 binding sites, the L2 (minor capsid protein) is also involved in the cell entry process. The current hypothesis suggests that the interaction between L1 and the primary cell receptor in vitro or HSPG in vivo promotes conformational changes on the virus's capsid. These changes reveal the N-terminus of L2 in the furin cleavage region, and they are speculated to reveal the binding site for the cell receptor [21]. Another outcome is the reduced affinity between the capsid and the HSPG and the subsequent transfer from the basement membrane to the keratinocyte [20]. The alpha6 integrin subunit has been suggested as the cell receptor but not prerequisite for the HPV cell infection [22] (Figure 1). As far as the uptake of the HPV is concerned, many patterns can be recognized depending on the HPV type. A common ground is the delayed kinetics of the process, which renders the virus susceptible to neutralization by antibodies [23]. The cell uses lysosome-associated membrane proteins to transfer the virus to the endosome where the uncoating of the virus begins about 12 h after the contact to the cell surface. L2 protein facilitates the escape from the endosome and the migration to the nucleus via the microtubule network, which is completed during the mitosis [24, 25]. L2 also disorganizes the nuclear domain 10, an accumulation of proteins which participate in transcriptional regulation, growth suppression, and apoptosis, probably in order to control cell cycle [26].

### 3.3. The Perfect Site

It is well known that the most vulnerable sites to tumorigenesis are where cell transformation occurs. Both cervix and anus belong to this category in contrast to vulva and vagina where no metaplasia occurs. The transformation zone (TZ) is the most common site of squamous intraepithelial lesion. Many researchers have tried to clarify the differences in the milieu and the immunosurveillance between the TZ and the exocervix. TZ is associated with a significant reduction in the density of Langerhans cells compared to the exocervix [27]. Additionally the expression of the immunosuppressive interleukin 10 (IL10) is more common in the TZ than in the exocervix [28].

### 3.4. The Uniqueness of HPV Interaction with Host Immune System

HPV viruses have a distinct capability to evade confrontation with human immune system. This important capability is achieved through three basic virus properties. Firstly, immune cells in the circulation cannot approach the virus easily since there is no viraemic phase. The initial infection is sited on the basement membrane whereas only Langerhans cells are abundant particularly in the apical layers of the mucosa. Secondly, HPV does not elicit major damage to the host cells, such as lysis of the infected cell, minimizing the inflammation and the subsequent signaling, allowing the virus to duplicate “silently” [29, 30]. The third evasion mechanism is the careful gene expression behavior of the virus. The expression of the oncogenes of the virus is kept at low levels throughout the initial life cycle, and highly immunogenic products, like L1, L2 capsid proteins, are synthesized only in superficial layers of the epithelium [31, 32].

### 3.5. Innate Immunity versus HPV

The innate immunity is the nonspecific part of the immune system. It is mediated by epithelial barrier, the complement system, and a variety of cells that phagocytose antigens and present them to other cells or destroy them.

The immunosurveillance of squamous epithelium of the cervix is managed by Langerhans cells (LCs), immature dendritic cells. LCs, which are abundant in the skin and mucosa, are considered to take up and process antigens in order to present them to the B and Tcells, eliciting both innate and adaptive immunity against the virus [33]. As mentioned before, in transformation zone compared to the exocervix, significantly decreased numbers of LCs are observed. In SILs (squamous intraepithelial lesions), a small increase in the density of LCs is observed but their function appears deficient [27, 34]. Dendritic cells recognize special patterns on the pathogens utilizing their Toll-like receptors and use major histocompatibility complex (MHC) to present the antigens to the Tcells, sometimes assisted by inflammatory agents such as chemokines and cytokines. However, even in absence of lesions, Langerhans cells of the epidermis do not produce a sufficient T-cell response, compared to the dendritic cells of the dermis, due to the lack of appropriate costimulatory microenvironment. Consequently, Langerhans cells may be unable to elicit a successful immune response and become a part of the virus tolerance tactics. Accordingly, potential vaccines should avoid using LC as presenting agent without using costimuli [35].

Toll-like receptors (TLRs) play a crucial role in innate immunity. They can be found on a variety of cells of innate immunity and recognize both endogenous and exogenous threats, specifically pathogen-associated molecular patterns (PAMP) and damage-associated molecular patterns (DAMP). The activation of TLRs elicits a proinflammatory expression profile which promotes innate immunity. The double stranded HPV DNA is recognized mainly by TLR 9, and a cascade of interferons (INF-*α*,  INF-*β*, and  INF-*γ*) is initiated [36]. TLRs and interferons pathways are targeted by HPV oncoproteins resulting in an aberrant expression pattern which contributes to the virus tenacity and carcinogenic potential [37]. The tumorigenic E6 and E7 genes in HPV 16 are responsible for the downregulation of TLR9, which is known to respond to DNA threats and evoke an innate immune reply [38]. Moreover, an increasing trend in TLR 3 expression, which usually recognizes RNA viruses, is observed in dysplastic epithelium [37]. Additionally INF-*κ*  and IL10 production appears disrupted in premalignant or malignant epithelium. INF-*κ* decreased expression is considered to be originated either from the methylation of INF-*κ*  promoter or the direct downregulation by the HPV oncogenes [39, 40]. Although TLRs and cytokines signaling is not yet completely clarified, the upregulation of certain TLRs is considered to be an attractive target for new treatments for cervical cancer [41, 42].

Macrophages are derived from monocytes and are situated in tissues. They belong to the phagocyte family and have a crucial part in both innate and initiating adaptive immune responses, by digesting pathogens and additionally stimulating lymphocytes and other immune cells. Certain proteins such as monocyte chemotactic protein-1 (MCP-1) and macrophage inflammatory protein (MIPa3) help the aggregation of macrophages. Both of these proteins appear downregulated directly or indirectly by HPV [43, 44]. Tumor environment observations have helped us see a different aspect of macrophage function. Accumulating evidence suggests that tumors are infiltrated by large amounts of macrophages which aggregate to the site due to the recognition of cancer cells as foreign cells [45]. Contrary to the predictable proinflammatory and antitumor functionality, macrophages inside the microenvironment of solid tumors can have a part in disease progression. Tumor associated macrophages (TAMs) promote cancer cell proliferation and migration, angiogenesis and restriction of immune defenses [46]. This can be explained by the identification of two distinct macrophage phenotypes: M1 proinflammatory and M2 immunomodulatory. M2 profile elicits an increased vascular endothelial growth factor (VEGF) and metalloprotease-9 in order to help in tissue repair, but when it is activated by tumor, it results in basement membrane disruption, tumor growth, and metastasis [45, 47, 48]. As cervical lesions progress, an increase in the number of macrophages is observed and M2 macrophages are the main population in HPV-associated tumors [49, 50]. M2 macrophages promote the differentiation of naïve T cells to T-regulatory cells through IL10 and consequently tumor expansion [50, 51]. Depletion of TAM can be considered as a possible target of immunotherapy.

Another important part of innate immune response against viral attack is attributed to natural killer cells (NK cells). NK cells are lymphocytes which respond quickly to stressed cells, either under viral attack or cancerous ones, without the need of major histocompatibility system (MHC). It was recently observed that in HSILs and cervical cancer by HPV16, the NK-activating receptors NKp30 and NKp45 (and NKG2D only in cancer) are considerably decreased, affecting the cytolytic functionality [52].

### 3.6. Adaptive Immunity versus HPV

Adaptive immunity, the specific immune response against the pathogens, consists of B cells and T cells. B cells are responsible for the humoral immune response and T cells, which are divided in helper T cells, cytotoxic T cells (CTLs), and regulatory T cells (Tregs), are responsible for a variety of functions.

T-helper cells, distinguishable due to the CD4 protein on their surface, set the cytokine milieu, determining the direction of the immune response. The conditions under which the mature, but immunologically virgin, T-helper lymphocytes are activated determine their phenotype and result in two distinct populations, Th1 and Th2. This differentiation between Th1 and Th2 is determined by a variety of factors such as the type of the antigen presenting cell, the existence of costimulating signals, the amount, the structure, and the entry point of the antigen, the duration and the repetitiveness of the antigen exposure, the presence of adjuvants, and the local microenvironment of cytokines and hormones. Both IFN-*γ* and IL12 are required for the differentiation of Th0 (naïve lymphocyte) to Th1, which produces IFN-*γ*, lymphotoxin  *α*, and IL2 (and IL10, TNF-*α*) and leads to the activation of cell mediated immunity. On the other hand for the Th2 phenotype, IL4 and IL2 are prerequisite, and the cytokine products consist of IL4, IL5, IL13, IL25, IL10, and amphiregulin, contributing to the development of humoral immune response [53, 54]. In general, after studying the unique pattern of T-cell response among women with different grades of cervical neoplasia, T-helper cells are suggested as the dominant response needed for an HPV lesion to be cleared [55]. The equilibrium between Th1 and Th2 must be sustained invariably in order to front intracellular or extracellular attacks. Nowadays, it is supported by a variety of studies that in HPV lesion the delicate balance between the two phenotypes is distorted. The HPV, as an intracellular enemy, should evoke Th1 immune response. However, it appears that in patients with intraepithelial and invasive cervical HPV lesions Th2 cytokine profile is prevalent. It is safe to note that reduced Th1 response and increased Th2 response lead to suppression of cellular immunity and lesion progression [56, 57]. IL2 and TNF-*α*  levels (both belong to the Th1 pattern) appear lower in HPV lesion than in healthy women [57, 58]. Although IL4 levels, characteristic Th2 product, seem to increase in LSIL, as the lesion progresses they decrease slightly. Overall, in HPV lesions both Th1 and Th2 phenotypes are suppressed, especially Th1, presumably due to the activity of the Treg cells.

The expression of CD8 glycoprotein on the surface of a T cell defines the cell as cytotoxic (CTL). CTL is the main agent in antigen specific immunity and recognizes the antigens with the assistance of MHC class I. Cell mediated immunity plays a pivotal role in clearance of HPV lesion. This is substantiated from the observation that HIV positive persons or patients who have undertaken chemotherapy after transplant, with a diminished T cell number, suffer from persistent HPV infections, either genital warts or intraepithelial lesions and cancer [59, 60]. The most T-cell activation is caused by HPV E6 and E7 proteins, and the destruction is assisted by the upregulation of adhesion molecules like ICAM-1, VCAM-1, and E-selectin in infected cells [61]. Nevertheless, HPV has developed defenses against cytotoxic cells. HPV E7 oncogene downregulates the expression of (antigen peptide transporter-1) TAP-1, which has an essential part in mounting MHC class I with the viral antigen, resulting in suppression in HPV's antigens presentation and offering HPV a great evasion tactic against human cellular defense [62, 63]. Additionally, it is noted that HPV16 E5 downregulates MHC/HLA class I [64].

T-regulatory cells are a subset of T cells expressing CD4, CD25, and transcription factor Foxp3, that is normally necessary in the induction of tolerance, but its increase may hinder the immune response and suppress antitumor defense through inhibiting cytokines. Their activation, which is induced by TGF-*β* and IL2, is considered as a poor prognostic for malignancy [65–67]. This Treg orchestrated suppression of immunity is still not completely clarified. It is presumed that Tregs reduce proliferation of T-helper and cytotoxic T cells. As for APCs, Tregs also distort their necessary protein expression of CD80 and CD86 and evoke the production of indoleamine 2.3-dioxygenase (IDO) by dendritic cells, which is an enzyme toxic to T-cell populations [68]. The role of TGF-*β* is still controversial, but it is considered a suppressive factor as it evokes the expression of Foxp3 in CD4+ cells and differentiates them to iT_reg_ cells (i = induced) [69, 70]. Other T_reg_ products like carbon monoxide, galectins, and IL10 pleiotropic activity in the tumor microenvironment should be further examined [71–73]. 

Recent studies have highlighted the part of Treg cell during the HPV infection. TGF-*β*1 and TGF-*β*2 levels are reported to increase as the lesion progresss from LSIL to invasive cervical cancer; in contrast IL12 and TNF-*α*  levels (classic Th1 pattern cytokines) drop significantly. IL10 is also rising as the disease deteriorates, especially in HSILs [57, 73].

B-cells are responsible for humoral response, which neutralize and opsonize viral agents. Humoral immunity is stimulated by antigen presenting cells and Th2 cytokine pattern and depends on CD4 helper T cells that assist B cells to mature and produce antibodies against a specific epitope. The antibodies against HPV target mainly the L1 capsid protein although weak antibodies directing against E2, E6, E7, and L2 have been described. The vast majority of these antibodies are IgG1 class, a predictable response against viral antigens [74]. There are two types of neutralizing L1 antibodies. The first class hinders cell surface binding while the second class prevents binding to the basement membrane. Both appear to prevent the viral internalization either by direct binding or by blocking necessary conformational changes. Eight to nine months after natural infection, sero-conversion and neutralizing antibodies can be estimated, but their levels are low and not apparent in all women [75].

The L1 neutralizing antibodies that are produced by virus-like particles (VLP) of prophylactic vaccination belonging to the second class are greater than those created in natural infection, and their serum levels remain high in long-term studies [32].

### 3.7. Therapeutic Vaccination: A New Era Awaits

Preventive vaccination against high risk HPV types 16 and 18 is of widespread use. Current vaccines utilize structural proteins L1 and L2 on virus-like particles [76, 77]. However, due to high cost and requirement for special preservation conditions they are not suitable for third world countries that suffer the most from HPV related disease [78]. It is assessed that first significant outcome on morbidity and mortality rates due to prophylactic vaccination will be apparent in 20 years at least. This assessment creates an imperative need for novel therapeutic endeavors, which will utilize the recent breakthroughs in understanding the immunological details of the infection.

A number of therapeutic vaccines are now in clinical trials all over the world. In contrast to preventive vaccines, therapeutic ones target the oncogenetic proteins E6 and E7 which are continuously expressed in the cells throughout the HPV infection. Furthermore, the requirement of these genes in cellular transformations makes them necessary for the virus [76]. Additionally, the L1, L2 expression appears to fade in HPV infected cells in which genome integration by high risk types has occurred.

On contrary to the stimulation of the humoral immunity aimed by the prophylactic vaccines, the therapeutic vaccines develop cell mediated immunity in order to control the infection.

An approach is the use of *viral or bacterial live vectors*, like *Listeria monocytogenes*, adenovirus, and vaccinia virus, which are highly immunogenic and broadcast the antigens to many APCs for process, stimulating activation of both CD4+ and CD8+ cells through MHC II and MHC I, respectively [79–86].

Peptide/protein based vaccines are safer and easier to produce, but their extracellular nature evokes primarily a humoral response, and they need adjuvants, such as cytokines, TLR ligands, and chemokines, in order to become adequately immunogenic [87–91].

Combination of both prophylactic and therapeutic vaccines is being developed, such as TA-CIN which contains a recombinant fusion protein incorporating HPV16 L2, E6, and E7 [92].

Another appealing approach is utilizing nucleic acid, in form of naked DNA or RNA replicon, repeatedly administrated, though they also need immunogenic amplification. The major targets are to increase the population of available APC cells by electroporation or protein-mediated intercellular transportation, to improve antigen expression and presentation in APCs, and to prolong APCs life span enabling them to prime more T cells [93–96].

The most complicated method of therapeutic vaccination appears to be the administration of autologous dendritic cells loaded with HPV antigens and ex vivo altered tumor cells enhanced with immunostimulatory proteins' genes [97, 98]. 

Combined venues with prime-boost regimens, such as subsequent vaccination, chemotherapeutic agents, like apigenin, and radiotherapy, have also been suggested [99–101]. The modulation of the formerly discussed tumor milieu, by targeting the Treg cells, is presumed as a hopeful perspective [102].

## 4. Conclusions

HPV infection is considered an epidemic and can lead to lethal cancers. Prevalence of cervical cancer and high mortality rates, especially in developing countries, are alarming and require our immediate attention. The complete comprehension of the immunological aspects of HPV lesion microenvironment, including human immunity components, HPV evasion, and defense tactics, is a significant stepping stone in the development of new preventive and therapeutic options. Effective innate and adaptive immune responses are necessary in order to beat the virus despite its impressive evasion tactics. Particularly, the clearance of HPV infections depends on a powerful Th1 polarized cell mediated immune response, constriction of Treg branch of immunity, and a clearly stimulating cytokine milieu. This cell mediated response is the main perspective of the developing vaccines. On contrary to preventive vaccines, new, therapeutic ones are destined to meliorate existing lesions and, consequently, the impact upon HPV-associated malignancies prevalence.

## References

[1] CDC (2012). *Epidemiology and Prevention of Vaccine-Preventable Diseases*.

[2] Jensen KE, Schmiedel S, Norrild B, Frederiksen K, Iftner T, Kjaer SK (2012). Parity as a cofactor for high-grade cervical disease among women with persistent human papillomavirus infection: a 13-year follow-up. *British Journal of Cancer*.

[3] Romero JIC, Girón CH, Marina VM (2011). Hormonal contraception as a risk factor for cancer prevention: biological evidence, immunological and epidemiological. *Ginecologia y Obstetricia de Mexico*.

[4] Luhn P, Walker J, Schiffman M (2013). The role of co-factors in the progression from human papillomavirus infection to cervical cancer. *Gynecologic Oncology*.

[5] Smith JS, Herrero R, Bosetti C (2002). Herpes simplex virus-2 as a human papillomavirus cofactor in the etiology of invasive cervical cancer. *Journal of the National Cancer Institute*.

[6] Mao C, Hughes JP, Kiviat N (2003). Clinical findings among young women with genital human papillomavirus infection. *American Journal of Obstetrics and Gynecology*.

[7] Ault KA (2006). Epidemiology and natural history of human papillomavirus infections in the female genital tract. *Infectious Diseases in Obstetrics and Gynecology*.

[8] Bernard H-U, Burk RD, Chen Z, van Doorslaer K, Hausen HZ, de Villiers E-M (2010). Classification of papillomaviruses (PVs) based on 189 PV types and proposal of taxonomic amendments. *Virology*.

[9] Smith JS, Lindsay L, Hoots B (2007). Human papillomavirus type distribution in invasive cervical cancer and high-grade cervical lesions: a meta-analysis update. *International Journal of Cancer*.

[10] Scheurer ME, Tortolero-Luna G, Adler-Storthz K (2005). Human papillomavirus infection: biology, epidemiology, and prevention. *International Journal of Gynecological Cancer*.

[11] Longworth MS, Laimins LA (2004). Pathogenesis of human papillomaviruses in differentiating epithelia. *Microbiology and Molecular Biology Reviews*.

[12] Zhang B, Spandau DF, Roman A (2002). E5 protein of human papillomavirus type 16 protects human foreskin keratinocytes from UV B-irradiation-induced apoptosis. *Journal of Virology*.

[13] Zur Hausen H (2002). Papillomaviruses and cancer: from basic studies to clinical application. *Nature Reviews Cancer*.

[14] Münger K, Howley PM (2002). Human papillomavirus immortalization and transformation functions. *Virus Research*.

[15] Faridi R, Zahra A, Khan K, Idrees M (2011). Oncogenic potential of human papillomavirus (HPV) and its relation with cervical cancer. *Virology Journal*.

[16] Joyce JG, Tung J-S, Przysiecki CT (1999). The L1 major capsid protein of human papillomavirus type 11 recombinant virus-like particles interacts with heparin and cell-surface glycosaminoglycans on human keratinocytes. *Journal of Biological Chemistry*.

[17] Giroglou T, Florin L, Schäfer F, Streeck RE, Sapp M (2001). Human papillomavirus infection requires cell surface heparan sulfate. *Journal of Virology*.

[18] Sapp M, Bienkowska-Haba M (2009). Viral entry mechanisms: human papillomavirus and a long journey from extracellular matrix to the nucleus. *FEBS Journal*.

[19] Culp TD, Budgeon LR, Peter Marinkovich M, Meneguzzi G, Christensen ND (2006). Keratinocyte-secreted laminin 5 can function as a transient receptor for human papillomaviruses by binding virions and transferring them to adjacent cells. *Journal of Virology*.

[20] Schiller JT, Day PM, Kines RC (2010). Current understanding of the mechanism of HPV infection. *Gynecologic Oncology*.

[21] Richards RM, Lowy DR, Schiller JT, Day PM (2006). Cleavage of the papillomavirus minor capsid protein, L2, at a furin consensus site is necessary for infection. *Proceedings of the National Academy of Sciences of the United States of America*.

[22] Evander M, Frazer IH, Payne E, Qi YM, Hengst K, McMillan NAJ (1997). Identification of the *α*6 integrin as a candidate receptor for papillomaviruses. *Journal of Virology*.

[23] Culp TD, Christensen ND (2004). Kinetics of in vitro adsorption and entry of papillomavirus virions. *Virology*.

[24] Selinka H-C, Giroglou T, Sapp M (2002). Analysis of the infectious entry pathway of human papillomavirus type 33 pseudovirions. *Virology*.

[25] Pyeon D, Pearce SM, Lank SM, Ahlquist P, Lambert PF (2009). Establishment of human papillomavirus infection requires cell cycle progression. *PLoS Pathogens*.

[26] Florin L, Schäfer F, Sotlar K, Streeck RE, Sapp M (2002). Reorganization of nuclear domain 10 induced by papillomavirus capsid protein l2. *Virology*.

[27] Giannini SL, Hubert P, Doyen J, Boniver J, Delvenne P (2002). Influence of the mucosal epithelium microenvironment on Langerhans cells: implications for the development of squamous intraepithelial lesions of the cervix. *International Journal of Cancer*.

[28] Mota F, Rayment N, Chong S, Singer A, Chain B (1999). The antigen-presenting environment in normal and human papillomavirus (HPV)-related premalignant cervical epithelium. *Clinical and Experimental Immunology*.

[29] Kanodia S, Fahey LM, Kast WM (2007). Mechanisms used by human papillomaviruses to escape the host immune response. *Current Cancer Drug Targets*.

[30] Stanley M (2006). Immune responses to human papillomavirus. *Vaccine*.

[31] Doorbar J (2005). The papillomavirus life cycle. *Journal of Clinical Virology*.

[32] Stanley M (2008). Immunobiology of HPV and HPV vaccines. *Gynecologic Oncology*.

[33] Manickam A, Sivanandham M, Tourkova I, Shurin M, Smolkin Y (2007). Immunological role of dendritic cells in cervical cancer. *Immune-Mediated Diseases*.

[34] Tay SK, Jenkins D, Maddox P (1987). Subpopulations of Langerhans’ cells in cervical neoplasia. *British Journal of Obstetrics and Gynaecology*.

[35] Fausch SC, Da Silva DM, Kast WM (2003). Differential uptake and cross-presentation of human papillomavirus virus-like particles by dendritic cells and Langerhans cells. *Cancer Research*.

[36] Akira S, Takeda K (2004). Toll-like receptor signalling. *Nature Reviews Immunology*.

[37] DeCarlo CA, Rosa B, Jackson R, Niccoli S, Escott NG, Zehbe I (2012). Toll-like receptor transcriptome in the HPV-positive cervical cancer microenvironment. *Clinical and Developmental Immunology*.

[38] Hasan UA, Bates E, Takeshita F (2007). TLR9 expression and function is abolished by the cervical cancer-associated human papillomavirus type 16. *Journal of Immunology*.

[39] Decarlo CA, Severini A, Edler L (2010). IFN-*κ*, a novel type i IFN, is undetectable in HPV-positive human cervical keratinocytes. *Laboratory Investigation*.

[40] Rincon-Orozco B, Halec G, Rosenberger S (2009). Epigenetic silencing of interferon-*κ* in human papillomavirus type 16-positive cells. *Cancer Research*.

[41] Mohan C, Zhu J (2010). Toll-like receptor signaling pathways—therapeutic opportunities. *Mediators of Inflammation*.

[42] So EY, Ouchi T (2010). The application of toll like receptors for cancer therapy. *International Journal of Biological Sciences*.

[43] Hacke K, Rincon-Orozco B, Buchwalter G (2010). Regulation of MCP-1 chemokine transcription by p53. *Molecular Cancer*.

[44] Guess JC, McCance DJ (2005). Decreased migration of Langerhans precursor-like cells in response to human keratinocytes expressing human papillomavirus type 16 E6/E7 is related to reduced macrophage inflammatory protein-3*α* production. *Journal of Virology*.

[45] Martinez FO, Helming L, Gordon S (2009). Alternative activation of macrophages: an immunologic functional perspective. *Annual Review of Immunology*.

[46] Mazibrada J, Rittà M, Mondini M (2008). Interaction between inflammation and angiogenesis during different stages of cervical carcinogenesis. *Gynecologic Oncology*.

[47] Quatromoni JG, Eruslanov E (2012). Tumor-associated macrophages: function, phenotype, and link to prognosis in human lung cancer. *American Journal of Translational Research*.

[48] Tang X, Mo C, Wang Y, Wei D, Xiao H (2013). Anti-tumour strategies aiming to target tumour-associated macrophages. *Immunology*.

[49] Hammes LS, Tekmal RR, Naud P (2007). Macrophages, inflammation and risk of cervical intraepithelial neoplasia (CIN) progression-Clinicopathological correlation. *Gynecologic Oncology*.

[50] Lepique AP, Daghastanli KRP, Cuccovia I, Villa LL (2009). HPV16 tumor associated macrophages suppress antitumor T cell responses. *Clinical Cancer Research*.

[51] Bolpetti A, Silva JS, Villa LL, Lepique AP (2010). Interleukin-10 production by tumor infiltrating macrophages plays a role in Human Papillomavirus 16 tumor growth. *BMC Immunology*.

[52] Garcia-Iglesias T, del Toro-Arreola A, Albarran-Somoza B (2009). Low NKp30, NKp46 and NKG2D expression and reduced cytotoxic activity on NK cells in cervical cancer and precursor lesions. *BMC Cancer*.

[53] Mosmann TR, Cherwinski H, Bond MW, Giedlin MA, Coffman RL (2005). Two types of murine helper T cell clone—I. Definition according to profiles of lymphokine activities and secreted proteins. 1986. *Journal of immunology*.

[54] Zhu J, Paul WE (2008). CD4 T cells: fates, functions, and faults. *Blood*.

[55] Steele JC, Mann CH, Rookes S (2005). T-cell responses to human papillomavirus type 16 among women with different grades of cervical neoplasia. *British Journal of Cancer*.

[56] Clerici M, Merola M, Ferrario E (1997). Cytokine production patterns in cervical intraepithelial neoplasia: association with human papillomavirus infection. *Journal of the National Cancer Institute*.

[57] Peghini BC, Abdalla DR, Barcelos AC, Teodoro L, Murta EF, Michelin MA (2012). Local cytokine profiles of patients with cervical intraepithelial and invasive neoplasia. *Human Immunology*.

[58] Kobayashi A, Weinberg V, Darragh T, Smith-McCune K (2008). Evolving immunosuppressive microenvironment during human cervical carcinogenesis. *Mucosal Immunology*.

[59] Chirgwin KD, Feldman J, Augenbraun M, Landesman S, Minkoff H (1995). Incidence of venereal warts in human immunodeficiency virus-infected and uninfected women. *Journal of Infectious Diseases*.

[60] Fruchter RG, Maiman M, Arrastia CD, Matthews R, Gates EJ, Holcomb K (1998). Is HIV infection a risk factor for advanced cervical cancer?. *Journal of Acquired Immune Deficiency Syndromes and Human Retrovirology*.

[61] Scott M, Nakagawa M, Moscicki A-B (2001). Cell-mediated immune response to human papillomavirus infection. *Clinical and Diagnostic Laboratory Immunology*.

[62] Vambutas A, DeVoti J, Pinn W, Steinberg BM, Bonagura VR (2001). Interaction of human papillomavirus type 11 E7 protein with TAP-1 results in the reduction of ATP-dependent peptide transport. *Clinical Immunology*.

[63] Keating PJ, Cromme FV, Duggan-Keen M (1995). Frequency of down-regulation of individual HLA-A and -B alleles in cervical carcinomas in relation to TAP-1 expression. *British Journal of Cancer*.

[64] Campo MS, Graham SV, Cortese MS (2010). HPV-16 E5 down-regulates expression of surface HLA class I and reduces recognition by CD8 T cells. *Virology*.

[65] Correll A, Tüettenberg A, Becker C, Jonuleit H (2010). Increased regulatory T-cell frequencies in patients with advanced melanoma correlate with a generally impaired T-cell responsiveness and are restored after dendritic cell-based vaccination. *Experimental Dermatology*.

[66] French JD, Weber ZJ, Fretwell DL, Said S, Klopper JP, Haugen BR (2010). Tumor-associated lymphocytes and increased FoxP3+ regulatory T cell frequency correlate with more aggressive papillary thyroid cancer. *Journal of Clinical Endocrinology and Metabolism*.

[67] Fu J, Xu D, Liu Z (2007). Increased regulatory T cells correlate with CD8 T-cell impairment and poor survival in hepatocellular carcinoma patients. *Gastroenterology*.

[68] Oderup C, Cederbom L, Makowska A, Cilio CM, Ivars F (2006). Cytotoxic T lymphocyte antigen-4-dependent down-modulation of costimulatory molecules on dendritic cells in CD4^+^ CD25^+^ regulatory T-cell-mediated suppression. *Immunology*.

[69] Nakamura K, Kitani A, Strober W (2001). Cell contact-dependent immunosuppression by CD4^+^CD25^+^ regulatory T cells is mediated by cell surface-bound transforming growth factor *β*. *Journal of Experimental Medicine*.

[70] Collison LW, Workman CJ, Kuo TT (2007). The inhibitory cytokine IL-35 contributes to regulatory T-cell function. *Nature*.

[71] Garín MI, Chu N-C, Golshayan D, Cernuda-Morollón E, Wait R, Lechler RI (2007). Galectin-1: a key effector of regulation mediated by CD4^+^CD25^+^ T cells. *Blood*.

[72] Lee SS, Gao W, Mazzola S (2007). Heme oxygenase-1, carbon monoxide, and bilirubin induce tolerance in recipients toward islet allografts by modulating T regulatory cells. *FASEB Journal*.

[73] Mocellin S, Marincola FM, Young HA (2005). Interleukin-10 and the immune response against cancer: a counterpoint. *Journal of Leukocyte Biology*.

[74] Viscidi RP, Schiffman M, Hildesheim A (2004). Seroreactivity to human papillomavirus (HPV) types 16, 18, or 31 and risk of subsequent HPV infection: results from a population-based study in Costa Rica. *Cancer Epidemiology Biomarkers and Prevention*.

[75] Carter JJ, Koutsky LA, Hughes JP (2000). Comparison of human papillomavirus types 16, 18, and 6 capsid antibody responses following incident infection. *Journal of Infectious Diseases*.

[76] Rose RC, Reichman RC, Bonnez W (1994). Human papillomavirus (HPV) type 11 recombinant virus-like particles induce the formation of neutralizing antibodies and detect HPV-specific antibodies in human sera. *Journal of General Virology*.

[77] Roden RBS, Ling M, Wu T-C (2004). Vaccination to prevent and treat cervical cancer. *Human Pathology*.

[78] WHO (2010). *Papillomavirus and Related Cancers in Europe*.

[79] Bermúdez-Humarán LG, Cortes-Perez NG, Lefèvre F (2005). A novel mucosal vaccine based on live lactococci expressing E7 antigen and IL-12 induces systemic and mucosal immune responses and protects mice against human papillomavirus type 16-induced tumors. *Journal of Immunology*.

[80] Zhou L, Zhu T, Ye X (2010). Long-term protection against human papillomavirus E7-positive tumor by a single vaccination of adeno-associated virus vectors encoding a fusion protein of inactivated E7 of human papillomavirus 16/18 and heat shock protein 70. *Human Gene Therapy*.

[81] Echchannaoui H, Bianchi M, Baud D, Bobst M, Stehle J-C, Nardelli-Haefliger D (2008). Intravaginal immunization of mice with recombinant *Salmonella enterica serovar typhimurium* expressing human papillomavirus type 16 antigens as a potential route of vaccination against cervical cancer. *Infection and Immunity*.

[82] Lin C-W, Lee J-Y, Tsao Y-P, Shen C-P, Lai H-C, Chen S-L (2002). Oral vaccination with recombinant Listeria monocytogenes expressing human papillomavirus type 16 E7 can cause tumor growth in mice to regress. *International Journal of Cancer*.

[83] Sewell DA, Douven D, Pan Z-K, Rodriguez A, Paterson Y (2004). Regression of HPV-positive tumors treated with a new Listeria monocytogenes vaccine. *Archives of Otolaryngology*.

[84] Sewell DA, Pan ZK, Paterson Y (2008). Listeria-based HPV-16 E7 vaccines limit autochthonous tumor growth in a transgenic mouse model for HPV-16 transformed tumors. *Vaccine*.

[85] Zurkova K, Babiarova K, Hainz P (2009). The expression of the soluble isoform of hFlt3 ligand by recombinant vaccinia virus enhances immunogenicity of the vector. *Oncology Reports*.

[86] Lee DW, Anderson ME, Wu S, Lee JH (2008). Development of an adenoviral vaccine against E6 and E7 oncoproteins to prevent growth of human papillomavirus-positive cancer. *Archives of Otolaryngology*.

[87] Cui Z, Huang L (2005). Liposome-polycation-DNA (LPD) particle as a carrier and adjuvant for protein-based vaccines: therapeutic effect against cervical cancer. *Cancer Immunology, Immunotherapy*.

[88] Einstein MH, Kadish AS, Burk RD (2007). Heat shock fusion protein-based immunotherapy for treatment of cervical intraepithelial neoplasia III. *Gynecologic Oncology*.

[89] Stewart TJ, Drane D, Malliaros J (2004). ISCOMATRIX*™* adjuvant: an adjuvant suitable for use in anticancer vaccines. *Vaccine*.

[90] Frazer IH, Quinn M, Nicklin JL (2004). Phase 1 study of HPV16-specific immunotherapy with E6E7 fusion protein and ISCOMATRIX*™* adjuvant in women with cervical intraepithelial neoplasia. *Vaccine*.

[91] Liu B, Ye D, Song X (2008). A novel therapeutic fusion protein vaccine by two different families of heat shock proteins linked with HPV16 E7 generates potent antitumor immunity and antiangiogenesis. *Vaccine*.

[92] Hibbitts S (2010). TA-CIN, a vaccine incorporating a recombinant HPV fusion protein (HPV16 L2E6E7) for the potential treatment of HPV16-associated genital diseases. *Current Opinion in Molecular Therapeutics*.

[93] Best SR, Peng S, Juang C-M (2009). Administration of HPV DNA vaccine via electroporation elicits the strongest CD8+ T cell immune responses compared to intramuscular injection and intradermal gene gun delivery. *Vaccine*.

[94] Klencke B, Matijevic M, Urban RG (2002). Encapsulated plasmid DNA treatment for human papillomavirus 16-associated anal dysplasia: a Phase I study of ZYC101. *Clinical Cancer Research*.

[95] Lin K, Doolan K, Hung CF, Wu TC (2010). Perspectives for preventive and therapeutic HPV vaccines. *Journal of the Formosan Medical Association*.

[96] Kim TW, Hung C-F, Boyd D (2003). Enhancing DNA vaccine potency by combining a strategy to prolong dendritic cell life with intracellular targeting strategies. *Journal of Immunology*.

[97] Ferrara A, Nonn M, Sehr P (2003). Dendritic cell-based tumor vaccine for cervical cancer II: results of a clinical pilot study in 15 individual patients. *Journal of Cancer Research and Clinical Oncology*.

[98] Santin AD, Bellone S, Roman JJ, Burnett A, Cannon MJ, Pecorelli S (2005). Therapeutic vaccines for cervical cancer: dendritic cell-based immunotherapy. *Current Pharmaceutical Design*.

[99] Fiander AN, Tristram AJ, Davidson EJ (2006). Prime-boost vaccination strategy in women with high-grade, noncervical anogenital intraepithelial neoplasia: clinical results from a multicenter phase II trial. *International Journal of Gynecological Cancer*.

[100] Chuang C-M, Monie A, Wu A, Hung C-F (2009). Combination of apigenin treatment with therapeutic HPV DNA vaccination generates enhanced therapeutic antitumor effects. *Journal of Biomedical Science*.

[101] Tseng C-W, Trimble C, Zeng Q (2009). Low-dose radiation enhances therapeutic HPV DNA vaccination in tumor-bearing hosts. *Cancer Immunology, Immunotherapy*.

[102] Chuang C-M, Hoory T, Monie A, Wu A, Wang M-C, Hung C-F (2009). Enhancing therapeutic HPV DNA vaccine potency through depletion of CD4^+^CD25^+^ T regulatory cells. *Vaccine*.

